# FAST CBT for pediatric OCD: A multiple-baseline controlled pilot trial of parent training in exposure and response prevention delivered *via* telehealth

**DOI:** 10.3389/fpsyg.2022.1009735

**Published:** 2022-12-13

**Authors:** Lara J. Farrell, Natalja A. Nabinger de Diaz, Sharna Mathieu, Matthew L. McKenzie, Taka Miyamoto, Caroline L. Donovan, Allison M. Waters, Sonja March, Nicole Bothma, Rianca Kroon, Gabrielle Simcock, Robert S. Ware, Robert R. Selles, Eric A. Storch, Thomas Ollendick

**Affiliations:** ^1^School of Applied Psychology & Griffith University Centre for Mental Health, Griffith University, South Port, QLD, Australia; ^2^School of Applied Psychology & Griffith University Centre for Mental Health, Griffith University, Mount Gravatt, QLD, Australia; ^3^Centre for Health Research & School of Psychology and Wellbeing, University of Southern Queensland (USQ), Darling Heights, QLD, Australia; ^4^Menzies Health Institute Queensland, Griffith University, South Port, QLD, Australia; ^5^Department of Psychiatry, University of British Columbia, Vancouver, BC, Canada; ^6^Department of Psychiatry and Behavioral Sciences, Baylor College of Medicine, Houston, TX, United States; ^7^Child Study Centre, Virginia Polytechnic University, Blacksburg, VA, United States

**Keywords:** CBT, ERP, child, adolescent, OCD, parent, telethealth

## Abstract

**Objective:**

The current study utilized a single case series, non-concurrent multiple baseline design to examine the efficacy of training parents *via* telehealth videoconferencing in exposure and response prevention (ERP) for home delivery of the treatment for their children and adolescents with obsessive compulsive disorder (OCD).

**Method:**

There were nine participants aged 8 to 14  years who had received a primary diagnosis of OCD. The design involved a series of AB replications, whereby following pre-treatment assessments participants were randomly assigned to either a 2-week (*n* = 4) or 3-week (*n* = 5) baseline condition with weekly monitoring of their child’s OCD symptoms. Following baseline, parents participated four weekly telehealth parent-training modules in delivering FAST (**F**amilies **A**ccessing **S**kills **T**raining) cognitive behavior therapy (CBT) with ERP (CBT-ERP) to children with OCD *via* videoconferencing with the clinician. Primary outcome measures were OCD symptom severity, diagnostic severity, and global functioning, which were assessed post-treatment and at 2 month follow-up.

**Results:**

The stability of the baseline period from pre-treatment to week 2 (for the 2-week condition) or to week 3 (for the 3-week condition) was established as there were no significant differences across baseline scores for parent target obsessions or parent target compulsions ratings. Significant improvements on the primary outcomes of clinician assessed symptom severity, diagnostic ratings, and global functioning were observed from baseline to post-treatment, and continued to 2 months follow-up.

**Conclusion:**

These data suggest that brief, parent training in FAST CBT-ERP *via* telehealth provides an overall effective intervention that is likely to be of most benefit to children and youth who are mild to moderate in severity.

## Introduction

Pediatric obsessive–compulsive disorder (OCD) is a debilitating, neurobehavioral disorder, frequently associated with profound impairments across multiple domains of a child’s life (Piacentini et al., 2003; Valderhaug and Ivarsson, 2005). Cognitive-behavioral therapy (CBT) with a focus on exposure and response prevention (ERP) is the most widely supported evidence-based psychosocial treatment for children with OCD, with large effect sizes from meta-analyses to support its efficacy (McGuire et al., 2015). Unfortunately, an estimated 60% of affected patients with OCD do not receive treatment (Kohn et al., 2004). Numerous barriers to accessing evidence-based care for OCD have been highlighted, including a lack of trained therapists, clinician and patient negative perceptions about CBT (e.g., reluctance to engage in exposure therapy; Young et al., 2014), geographical and financial barriers, and the time intensive nature of traditional weekly therapy (Goisman et al., 1993; Turner et al., 2009; Marques et al., 2010). One approach to improving access to care is *via* more efficient models of treatment delivery that maximize reach, while maintaining integrity and efficacy.

Internet delivered CBT (ICBT) has the potential to improve reach and provide a cost-effective alternative to therapist delivered face-to-face therapy. Lenhard et al. (2018) developed an ICBT program for childhood OCD, which involved 12 sessions of CBT delivered *via* the internet with brief therapist assistance to guide families through the program. In the first randomized controlled trial (RCT), 67 12-to 17-year-old adolescents with OCD were assigned to a 12-week therapist assisted ICBT or a waitlist (WL) condition. ICBT was found to be superior to the WL, with large effect size in favor of ICBT (between groups *d* = 0.69). However, despite this effect size, only 27% of youth in ICBT were classified as responders at post-treatment, which is considerably lower than the average response rates across face-to-face CBT trials (i.e., 68%, (McGuire et al., 2015). In a subsequent RCT of stepped-care ICBT, 54% of youth who received ICBT were considered responders at 3 months follow-up, relative to 71% who received face-to-face CBT (Aspvall et al., 2021). While ICBT offers promise in increasing reach, it does not appear to achieve the more favorable response rates observed in face-to-face therapy. Indeed, there may be a lower response to ICBT as patients do not always complete a full multicomponent online CBT program (Hadjistavropoulos et al., 2017), thus they may be missing out on the EPR treatment component. Dismantling studies of CBT for pediatric OCD have highlighted that ERP may be the most important component of CBT to achieve good response (Ale et al., 2015) and therefore, efficacy of ICBT may be enhanced by ensuring ERP is received.

Recently, cumulative research has also demonstrated the effectiveness of brief, intensive CBT formats for child anxiety and OCD (Öst and Ollendick, 2017), which delivered the core components of CBT (i.e., exposure therapy) in a more time efficient manner to increase reach and cost-effectiveness. Our team has developed an intensive ERP treatment for pediatric OCD (2–3 sessions; Farrell et al., 2016, 2022) that has demonstrated efficacy among young people with moderate to severe OCD, despite substantive reduction in therapy time. In an initial controlled multiple baseline case series (*n* = 10, Farrell et al., 2016), the efficacy of the intensive treatment, involving one psychoeducation session, two sessions of 3-h ERP plus telehealth maintenance delivered once per week for 3-weeks (*via* videoconferencing), was examined across parent–child- and clinician-rated measures at post-treatment and 6-month follow-up. Overall, there were significant reductions in OCD severity, diagnostic severity and improvements in quality of life, with the majority of the sample (80%) considered reliably improved, and resulting in clinically significant change. At post-treatment, 80% were considered responders, and 60% were in remission of symptoms, the latter increasing to 70% at 6-month follow-up. In a more recent large RCT of d-Cycloserine augmented intensive ERP (three sessions of 3-h ERP) for pediatric OCD relative to placebo controlled intensive ERP (*n* = 100, children 7 to 17 years), there were significant reductions in OCD severity and diagnostic severity, and improvements in functioning, for youth across both conditions following intensive ERP (Farrell et al., 2022). At 6-month follow-up, between 69% and 74% of youth were responders (based on conservative responder criteria, Farhat et al., 2021) and 49%–51% were in remission following intensive ERP (Farrell et al., 2022). Thus, there is good empirical support for brief, intensive ERP for pediatric OCD (Storch et al., 2007; Whiteside et al., 2014; Riise et al., 2016; Canavera et al., 2022; Farrell et al., 2022), which could improve access to specialized treatment further if it could be delivered in even more efficient modalities, such as telehealth videoconferencing.

Furthermore, given the increased tolerability of online delivered CBT and recognition that a full multicomponent intervention may not provide sufficient ERP for symptom response, one approach to reduce the research-service gap is to draw from the strengths of each of these approaches and deliver intensive ERP approaches *via* telehealth. Recognizing that children and youth with OCD require encouragement and support in the implementation of ERP at home, brief parent training in ERP *via* telehealth may provide an opportunity to increase the reach of evidence based ERP, while maintaining efficacy. Parent involvement in implementation of CBT for pediatric OCD has been demonstrated in a RCT of CBT with young children (e.g., POTS Jr.) with children aged 5 to 8 years (Freeman et al., 2014) where parents were explicitly and systematically trained in the core components of CBT and actively involved in supporting implementation. Given that OCD symptoms are frequently worse in the home environment (Valderhaug and Ivarsson, 2005) and impact markedly on parents *via* family accommodation (Storch et al., 2007; Peris et al., 2008; Stewart et al., 2008); harnessing the power of “parents as ERP therapists” presents a novel approach to reducing OCD symptoms, and equipping parents with the skills, when and where they need them, to manage child OCD symptoms and support longer-term generalization and maintenance.

The current study therefore aimed to test the preliminary efficacy of brief, parent training in ERP for parents of children and adolescents with OCD delivered by the therapist *via* telehealth (video-conferencing sessions over the internet). The treatment—FAST CBT (**F**amilies **A**ccessing **S**kills **T**raining in CBT-ERP), involved four parent-training modules in delivering ERP to children with OCD. Directly training parents in delivering ERP for treatment of pediatric OCD has not previously been investigated, and therefore FAST CBT represents a novel treatment for OCD.

A multiple baseline controlled design was used. This design is supported by the evidence-based treatment movement (Task Force on Promotion and Dissemination, 1995) and allows for the systematic evaluation of the efficacy of innovative treatments in a controlled manner (Jarrett and Ollendick, 2012; Oar et al., 2015) Children and youth (aged 8 to 14 years) with a primary diagnosis of OCD, along with their parent, were randomly assigned to a 2-or 3-week baseline monitoring condition followed by the telehealth delivered parent training intervention. It was hypothesized that OCD targeted symptoms (rated by parents) would remain stable during the baseline periods and then improve significantly following telehealth-delivered parent training in ERP. Moreover, it was predicted that significant improvements would be observed from pre-to post-treatment on primary outcomes of clinician ratings of OCD severity, diagnostic status, and global functioning. Further, it was hypothesized there would be significant improvements on secondary outcomes of child internalizing and externalizing symptoms, family accommodation of OCD, and self-reported parental depression, anxiety and stress symptoms. Finally, it was expected that post-treatment gains would be maintained at 2-month follow-up.

## Materials and methods

### Participants

Prospective participants were recruited from the community (South East Queensland, Australia) *via* social media announcements and by referrals into the program by general practitioners and local schools. Potential participants were initially screened (*N* = 20) for inclusion on the basis of presence of OCD symptoms. Inclusion criteria for enrolment into the study were (1) child aged 7 to 17 years with a DSM-5 (American Psychiatric Association, 2000) primary diagnosis of OCD; (2) at least one parent willing to attend e-therapy sessions; and (3) if a child was on medication for OCD, their medication dose was stable for 12 weeks prior to enrolment and remained stable for the duration of the trial. Exclusion criteria included psychosis, intellectual disability, or receiving concurrent psychotherapy. There were no referrals to the project during this time that met exclusion criteria. Children who were excluded due to not meeting inclusion criteria (i.e., the absence of sufficient OCD symptoms, or where OCD symptoms were not the main focus of the referral) were referred elsewhere (*n* = 7). Four eligible families declined to participate in favor of seeking child-focused therapy.

Participants were nine children and adolescents (aged 8 to 14 years), with a mean age of 11.67 years (SD = 2.06), comprised of three males and six females. In all cases mothers were the parent participating (except in one case where both mother and father were participants in the treatment), with mothers having a mean age of 45.88 years (SD = 6.20). All participants were of Caucasian ethnicity. One child was living in a single parent household with the biological mother (*n* = 1), all other children were living in a two-parent household with both of their biological parents (*n* = 6) or with the biological mother and stepfather (*n* = 1). Based on diagnostic interviews (ADIS-P; Silverman et al., 2022), this sample was deemed within the moderate to severe range of severity for OCD (*M* = 5.67, SD = 1.12 on a 0–8 scale), and consisted of high comorbidity, with 78% presenting with secondary and tertiary psychiatric diagnoses, and 33% holding a fourth diagnosis. In all cases, OCD was the primary reason for referral. Overall, children presented with nil to five comorbid diagnoses, in addition to their OCD (*M* number of comorbid diagnoses = 3.71, SD = 1.15). Comorbidity included other anxiety disorders, attention-deficit/hyperactivity disorder (AD/HD), autism spectrum disorder (ASD), and Tourette’s Syndrome. Two children (22%) were stabilized on SSRI medication for OCD at enrolment. Table 1 presents diagnostic information for the sample, OCD severity and medication status, as well as family income.

**Table 1:** Participant characteristics.

Participant	Age range (yrs.mths)^a^	Gender	Medication	Family income	CY-BOCS pre score	OCD Diagnosis CSR	Secondary diagnosis	Tertiary diagnosis	Total number of comorbid diagnoses
1	8.0–9.11	F	-	>100 K	19	6	GAD	AD/HD	2
2	10.0–11.11	M	-	60–70 K	26	4	Specific Phobia	-	1
3	10.0–11.11	M	Fluoxetine	60–70 K	29	7	Social Phobia	Specific Phobia	2
4	12.0–13.11	F	-	>100 K	24	5	ASD	Tourette’s	2
5	12.0–13.11	M	-	Unknown	25	7	Specific Phobia	GAD	5
6	10.0–11.11	F	-	50–60 K	22	4	-	-	0
7	12.0–13.11	F	-	>100 K	26	6	GAD	Specific Phobia	3
8	14.0–16.11	F	-	>100 K	29	6	GAD	Social Phobia	4
9	14.0–16.11	F	Sertraline	>100 K	28	6	-	-	0

a Age range was used to avoid indirectly identifiable data.

M = Male; F = Female; K = 1,000x; CYBOCS = Child Yale-Brown Obsessive–Compulsive Scale; CSR = Clinician Severity Rating as per ADIS; OCD = Obsessive Compulsive Disorder; GAD = Generalized Anxiety Disorder; ASD = Autism Spectrum Disorder; AD/HD = Attention-Deficit/Hyperactivity Disorder.

### Design

The present study evaluated the effectiveness of a parent-focused telehealth intervention aimed at training parents of young people with OCD to deliver ERP at home. The study utilized a single case series, non-concurrent multiple baseline design (Kazdin, 1998; Hayes et al., 1999). This involved a series of AB replications, whereby following pre-treatment assessment, participants were randomly assigned to either a 2-week baseline condition (*n* = 4), or a 3-week baseline condition (*n* = 5), using a computer-generated list of randomly permuted blocks. Treatment consisted of 4 weekly 1-h modules with parents (telehealth delivery); following by a maintenance phase of brief 15 min phone calls for 3 weeks to support exposure implementation. Assessments occurred at pre-treatment (baseline, week 0), mid-treatment (immediately following the 4 weekly parent module sessions, week 5), post treatment (following maintenance phase, week 8) and two-month follow-up (week 12). The framework that guided our treatment approach was informed by inhibitory learning models of ERP (Jacoby and Abramowitz, 2016).

## Measures

### Primary outcome measures

#### The anxiety and related disorders interview schedule for DSM-5—parent version, with autism spectrum addendum (ADIS/ASA—P)

The ADIS-C/P was developed specifically to diagnose anxiety disorders and commonly occurring comorbidity in children (Silverman and Eisen, 1992) and possesses good inter-rater and retest reliability (Silverman et al., 2022). The ADIS-C/P has demonstrated good sensitivity to treatment effects in both childhood anxiety (Kendall, 1994; Barrett et al., 1996; Ollendick et al., 2009) and OCD research (Knox et al., 1996; Barrett et al., 2004). This interview was administered to the child’s parent/s over the telephone. The ADIS-C/P has been shown to be as reliable when administered over the phone as when delivered face to face (Rapee et al., 1994). Each diagnosis receives a Clinician Severity Rating (CSR) based on clinician judgment, scored 0–8, with a score of 4 and above indicating a clinically significant diagnosis. Independent inter-rater reliability of ADIS-P interviews and CSR ratings by our trained assessors have been previously, and consistently, established as excellent (i.e., primary diagnosis *κ* = 1.0; secondary diagnosis *κ* = 0.84–1.0; tertiary diagnosis *κ* = 0.83–1.0; see Farrell et al., 2012,^2013^).

#### Children’s Yale-Brown Obsessive–Compulsive Scale (CY-BOCS)

The CY-BOCS is a clinician-rated, semi-structured interview, assessing severity of OCD symptomatology (Scahill et al., 1997). The CY-BOCS rates severity of obsessions and compulsions across five scales: (a) time occupied, (b) interference, (c) distress, (d) resistance, and (e) degree of control, and also provides a total severity score. The CY-BOCS shows reasonable reliability and validity, with good to excellent inter-rater agreement for the total score, as well as the obsessions and compulsions subscales (*r* = 0.84; 0.91; and 0.66; respectively) and high internal consistency for the total score (*α* = 0.87; Scahill et al., 1997). Independent research groups have also provided support for the scale’s psychometric properties for use among children and adolescents (Storch et al., 2004; Yucelen et al., 2006; Gallant et al., 2008). This interview was administered to children and parents together to assess overall OCD symptom severity.

#### Children’s Global Assessment Scale (CGAS)

The CGAS provides a single score of a child’s overall global functioning (1 = poorest functioning to 100 = highest functioning) and captures important clinical information beyond diagnostic categories (Shaffer et al., 1983). Functioning was reviewed during assessment supervision with the lead author (LJF) and determined based upon the overall assessment results of the ADIS-P, CY-BOCS and clinical impression.

### Secondary outcome measures

#### Child behavior checklist for ages 6–18 (CBCL/6–18)

The CBCL/6–18 is a parent report measure comprising of 113 items used to assess a range of emotional and behavioral problems in children (Achenbach and Rescorla, 2016). A 3-point scale is used to rate items (ranging from 0 = never true, 1 = sometimes true, 2 = often/always true). The CBCL yields internalizing and externalizing problems indices, which were used for analyses in this study. Research studies have demonstrated robust psychometric properties for the CBLC/6–18 (Heubeck, 2000; Aschenbrand et al., 2005), and extensive normative data are available for children aged 6 to 18 (Achenbach and Rescorla, 2016).

#### Depression Anxiety and Stress Scale-21 (DASS-21)

The DASS-21 is a 21 item self-report measure used to assess the severity and frequency of depression, anxiety, and stress symptoms in adults (Lovibond and Lovibond, 1995). The measure has three sub-scales, Depression, Anxiety and Stress, with seven items per scale, which are calculated by summing the scores across respective items. Respondents rate the extent to which they experience each symptom on a 4-point scale (ranging from 0 = not at all, to 3 = very much). Psychometric analyses of the DASS-21 support good reliability, construct and convergent validity (Lovibond and Lovibond, 1995; Anthony et al., 1998). In this study, the DASS-21 was used to obtain self-reported depression, anxiety, and stress experienced by parents receiving skills training.

#### Family Accommodation Scale 13 (FAS13)

The FAS13 is a parent-rated measure consisting of 13 items concerning parental accommodation to a child’s OCD (Calvocoressi et al., 1995). The extent that family members have accommodated the child’s OCD during the previous month (nine items) and the degree to which family members and the child experienced distress due to accommodating or not accommodating the child (four items) are assessed. Items are scored on a 5-point Likert scale (ranging from 0 = never/no accommodation, to 4 = daily/extreme accommodation), and summation of these items results in a total score. Higher scores have been associated with greater familial stress and symptom severity, and decreased family functioning (Calvocoressi et al., 1995). The FAS13 has good psychometric properties, such as internal consistency for the FAS total score (alphas ranging from.76 to.80; (Calvocoressi et al., 1995), good inter-rater reliability (Calvocoressi et al., 1999), and good convergent and divergent validity (Flessner et al., 2011).

### Baseline monitoring measure

#### Parent rated target symptoms—obsession and compulsions (TS obsession, TS compulsion)

Three individualized target obsessions (TS obsessions) and three target compulsions (TS compulsions) were obtained for each child, which represented symptoms of highest clinical significance for the family, derived from the CY-BOCS assessments. Target symptoms were rated by the parent/s indicating the child’s level of distress/difficulty associated with each symptom (e.g., obsession of harm coming to parents; compulsion of lengthy bedtime ritual) on a scale ranging from 0 to 8 (*how fearful/difficult?* 0 = none to 8 = very, very much). Target symptom ratings were averaged across the three symptoms to provide a mean target symptom obsession and target symptom compulsion score (Oar et al., 2015; Farrell et al., 2018).

### Procedure

**Figure 1:**
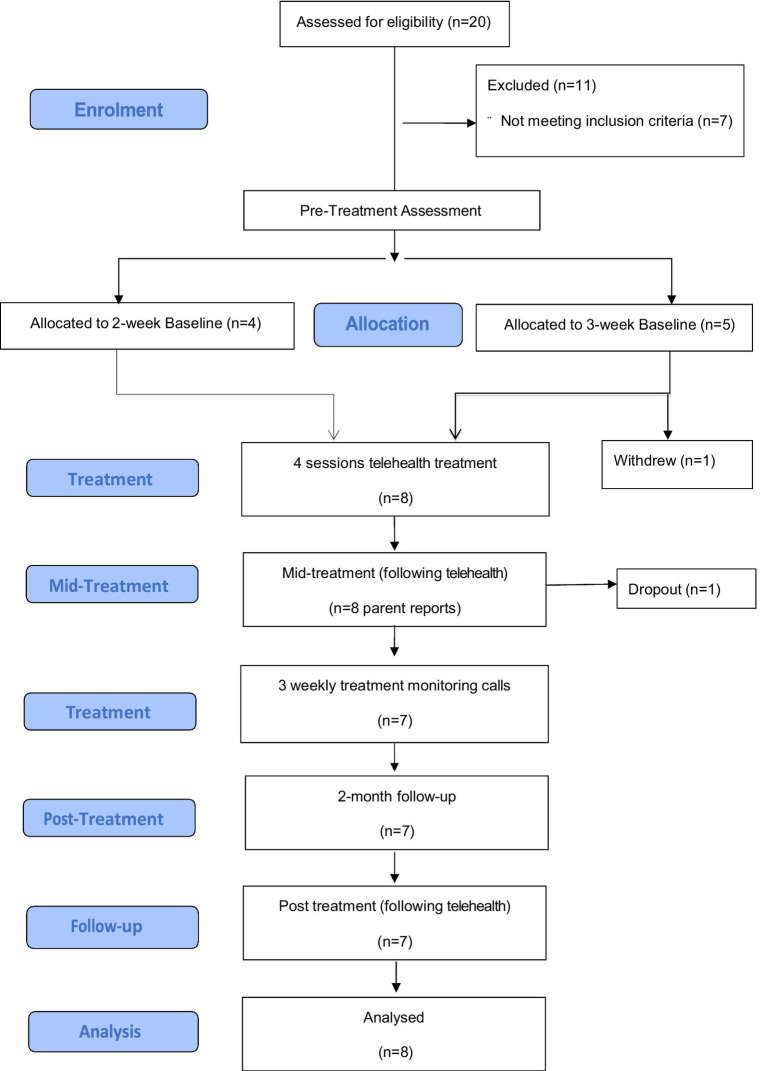
Participant flow diagram.

Following full ethical approval, the study was advertised in the community to assist in recruitment. Interested parents completed an initial brief telephone screen in order to assess their child’s eligibility (refer Figure 1). If the child was deemed suitable, the parent was emailed study information and consent forms and then an appointment was scheduled to complete the telephone delivered ADIS-IV-P (Lyneham and Rapee, 2005). Children deemed appropriate were invited to complete the CY-BOCS interview with their parents *via* e-therapy, and then parents completed various self-report measures.

### Assessment and treatment clinicians

The postgraduate-level clinicians reviewed diagnostic profiles with the supervising clinical psychologist (LJF) in order to determine final consensus diagnoses and confirm eligibility to proceed to the next stage of the study. At the 2-month follow-up and at the mid-treatment assessment the diagnostic interviews were conducted *via* telephone/telehealth by independent raters, who were blind to previous assessment information and who did not deliver the intervention. Treatment integrity was ensured by clinicians being trained in the protocol (LJF) and by following a protocol developed for the purpose of this study.

### Baseline monitoring, post intervention assessments

Families were randomized 1:1 to either a two-week baseline or a three-week baseline phase following a computer-generated randomization. On a weekly basis during the baseline period (either 2 or 3 weeks), parents completed telephone-administered ratings of the target symptoms (obsessions and compulsions). At post-treatment (week 8), parents completed diagnostic interviews (e.g., ADIS-P) over the telephone, and parents and children completed the CY-BOCS severity interview *via* telehealth videoconference. Parents also completed the target symptoms at the post-treatment ADIS-P interview, and completed an online survey of secondary outcomes measures at this time-point. At mid-treatment (week 5) and 2-month follow-up (week 13), diagnostic interviews, CGAS ratings and CY-BOCS interviews were again conducted *via* telephone/telehealth by blinded independent raters, who were blind to previous assessment information and who did not deliver the intervention. Parent ratings of target symptoms were also obtained.

### Intervention

#### Parent and child education session *via* telehealth

Upon completion of the baseline monitoring phase the children and parents attended an individual one-hour psychoeducation session, delivered by a clinician *via* telehealth videoconferencing. They received psychoeducation about OCD, including information about the development of OCD, common symptoms, and course of the disorder. Moreover, they were presented with education on the nature of CBT for childhood OCD, with a specific focus on the principles of ERP. The brief, concentrated nature of the treatment was discussed with the parent and child and a rationale for this approach was provided. Specifically, therapists ensured the child and parent understood that the parent training sessions (four weekly telehealth modules) were designed to provide a “kick start” to bossing back OCD, and that the parent and child would need to continue to practice facing and fighting OCD (ERP) during the month following the parent training sessions (maintenance phase). The therapist also discussed the role of family accommodation in OCD and how accommodation practices serve to maintain and worsen OCD symptoms over time. Parents and children were introduced to contingency management strategies in order to reward their child for exposure practice.

#### Parent training *via* telehealth

Prior to commencement of the parent training modules, parents were emailed handouts that comprised information and worksheets relevant to each module. The parent training program consisted of four 1-h modules which the therapist delivered *via* videoconferencing to the parents weekly over 4 weeks. The four modules specially trained parents in delivering ERP at home and included setting weekly homework for ERP implementation by parents with their child or adolescent. The modules included (1) *psychoeducation on ERP*—why ERP is used and how ERP helps to boss back OCD; (2) *facing and fighting plans*—developing fear hierarchies to begin facing (exposure) and fighting (response prevention) OCD; (3) *OCD Busting*—how to implement ERP at home and across contexts to maximize success, including contingency management principles; (4) *overcoming obstacles*—tackling common obstacles to ERP implementation (including parent, child, contextual challenges).

#### Maintenance program

After completion of the four parent training sessions, families completed a 3-week maintenance phase. The therapist telephoned the parent once a week (approximately 15 min per call) to review ERP progress over the past week, and problem solved any difficulties with completing the agreed ERP homework tasks. The parent and therapist then collaboratively decided upon ongoing ERP tasks for the following week. During the final telehealth session, relapse prevention was discussed.

### Overview of analyses

Single-case data were examined *via* visual inspection of the participants’ ratings across baseline, treatment and follow-up periods in line with recent guidelines for reporting single-case data (i.e., SCRIBE Statement; Tate et al., 2016). Stability over the baseline was examined by way of repeated measures ANOVA (for 2-and 3-week baseline). A series of repeated measures ANOVAs were then conducted followed by component pairwise comparisons, to examine participant changes over time on the primary outcome measures (CSR, CY-BOCS, CGAS) and secondary outcome measures (CBCL internalizing, CBCL externalizing, FAS13, DASS-21). A Reliable Change Index (RCI; Jacobson and Truax, 1991) was calculated to determine whether the magnitude of change in children’s OCD severity (CY-BOCS) was statistically reliable. An RCI cut-off of 1.96 standard deviation units was used to meet criteria for reliable improvement. Clinically significant improvement, defined by Jacobson and Truax (1991) as a change of two standard deviations from the pre-treatment mean, was also assessed in relation to OCD symptom severity (CY-BOCS) and OCD diagnostic severity (CSR). “Response” was reported at post-treatment and 2-month follow-up, and defined as a reduction of ≥35% on CY-BOCS severity, whereas “remission” at post-treatment or follow-up was a CY-BOCS of ≤12 (Storch et al., 2010).

## Results

### Participant retention

All nine families completed the parent and child pre-intervention phone assessments. Of the initial nine families, eight families completed the baseline monitoring and the first treatment phase (four parent-focused telehealth treatment sessions); one family withdrew interest during the baseline phase, due to competing family demands hindering their capacity to fully engage in treatment (Participant 5). Of the remaining eight families, one child withdrew during the mid-module assessment (Participant 4, parent OCD diagnostic module only completed), and then the family subsequently withdrew to pursue child-focused intervention instead. Thus, seven families completed the mid-module assessments following the each of the four weekly telehealth sessions. All remaining seven families completed the three therapist weekly maintenance monitoring telephone calls, and 2-month follow-up telephone assessments. Due to incomplete baseline data and early withdrawal, Participant 5 was excluded from all analyses (*n* = 8), while an intention to treat approach was utilized for all other data and last observation carried forward (LOCF) was used for Participant 4 with the pre scores carried forward on all measures except for the OCD CSR (carried from mid-treatment).

### Primary outcome measures

**Figure 2:**
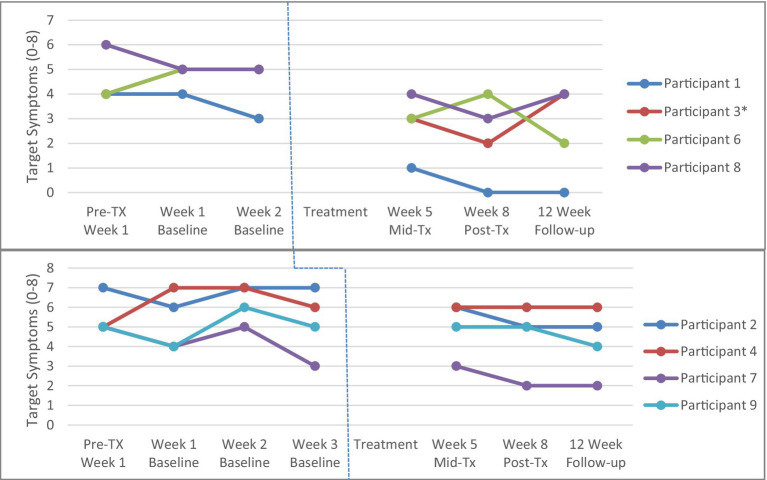
Parent Target Obsessions across 2-week (top panel, *n* = 4) and 3-week (bottom panel, *n* = 4) baseline conditions. Tx = treatment *Participant 3 and Participant 6 have identical scores so the red line for Participant 3 is not visible. Participant 4 did not complete the mid-, post-treatment and 2-month follow-up assessments, and therefore the last observation is taken from the week 3 baseline and carried forward.

**Figure 3:**
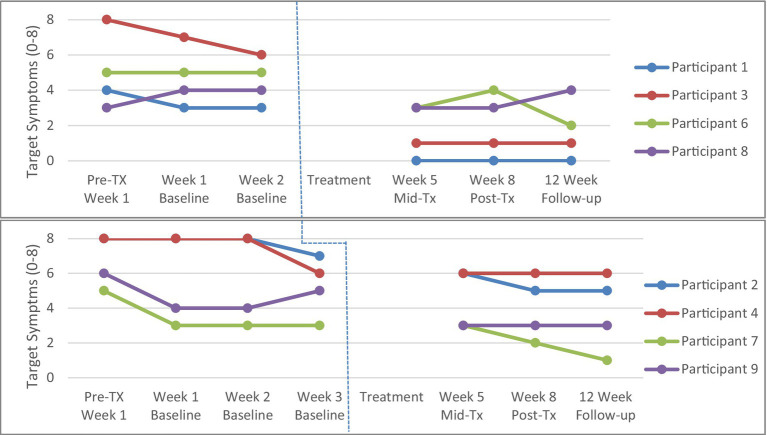
Parent Target Compulsions across 2-week (top panel, *n* = 4) and 3-week (bottom panel, *n* = 4) baseline. Tx = treatment. Participant 4 did not complete the mid-, post-treatment and 2-month follow-up assessments, and therefore the last observation is taken from the week 3 baseline and carried forward.

To establish the stability of the baseline period from pre-treatment to week 2 (for the 2-week condition) or to week 3 (for the 3-week condition), analyses were conducted separately for the two-week (*n* = 4) and three-week (*n* = 4) baseline groups using repeated measures ANOVAs. There were no significant differences across baseline scores (i.e., 1 week, 2 weeks, and 3 weeks) for parent target obsessions (*p* > 0.05; see Figure 2) or parent target compulsions ratings (*p* > 0.05; see Figure 3).

Across primary outcome measures (see Table 2), there were significant within-subjects repeated measures effects for time, with reductions from pre-treatment to post-treatment and 2-month follow-up on CSR ratings *F*(3, 21) = 4.13, *p* = 0.019; CY-BOCS total severity *F*(3, 21) = 8.30, *p* < 0.001; and CGAS *F*(3, 21) = 10.45, *p* < 0.001, with no differences between intention to treat (presented here) and completer data analyses (Table 1; *n* = 7) except for higher effect sizes when examining completers only. Effect sizes (η_p_^2^) are presented in Table 2.

**Table 2:** Means, standard deviations, and time main effects for intention to treat treatment outcome measures (*N* = 8).

Measure	Pre-treatment	Mid-treatment	Post-treatment	2-month F/up	Significance (*p*)	Effect size *ηp*^2^ (*d*)*
CSR	5.50 (1.07)	4.25 (1.98)	3.63 (2.39)	3.00 (2.20)	0.019*	0.371 (*1.13*)
CY-BOCS total score	25.38 (3.54)	19.63 (7.58)	19.13 (4.97)	17.13 (5.96)	<0.001***	0.543 (*1.86*)
CGAS	56.25 (6.41)	65.63 (12.08)	66.88 (11.93)	69.38 (10.16)	<0.001***	0.599 (*−1.86*)
CBCL Internalizing	16.38 (9.14)	-	12.00 (6.30)	-	0.020 *	1.07
CBCL Externalizing	9.88 (8.37)	-	6.75 (5.92)	-	0.104	0.66
FAS13	22.25 (6.54)	-	13.38 (6.28)	-	0.004 **	1.51
DASS21	10.75 (6.43)	-	6.75 (4.46)	-	0.105	0.66

*p < 0.05; **p < 0.005; ***p < 0.001; CSR = ADIS-P Clinician Severity Rating; CY-BOCS = Children’s Yale Brown Obsessive Compulsive Scale; Cohen’s (*d*) calculated based on paired samples *t*-tests, either pre to 2 months follow-up, or pre-to post in the case of CBCL internalizing and externalizing, FAS13, and DASS 21.

A significant reduction in CSR was found from pre-to post-treatment *t*(7) = 2.71; *p* = 0.030, and pre-treatment to 2-months *t*(7) = 3.21, *p* = 0.015, however, there was no significant change from pre-to mid-treatment, or from post-treatment to 2-month follow-up (*p* > 0.05). There was a significant reduction in CY-BOCS total scores from pre-to mid-treatment *t*(7) = 2.64; *p* = 0.033, pre-to post-treatment *t*(7) = 3.66; *p* = 0.008, and pre-treatment to 2-months *t*(7) = 5.27; *p* = 0.001, and no significant change from post-treatment to 2-month follow-up (*p* > 0.05). Finally, on the CGAS, there was a significant increase in scores from pre-to mid-treatment *t*(7) = −3.23; *p* = 0.014, pre-to post-treatment *t*(7) = −4.12; *p* = 0.004, and pre-treatment to 2-months *t*(7) = −5.27; *p* = 0.001, and no significant change from post-treatment to 2-month follow-up (*p* > 0.05).

Across secondary outcome measures assessed at baseline and post-treatment (see Table 2), there was a significant decrease in parent-reported child internalizing symptoms (CBCL) *t*(7) = 3.02; *p* = 0.02, and parents’ self-reported family accommodation (FAS13) *t*(7) = 4.28; *p* = 0.004 from pre-to post-treatment. However, there was no change on parent-reported child externalizing symptoms (CBCL) or parents’ depression, anxiety and stress symptoms (DASS21; *p* > 0.05). Effect sizes (*d*) from pre-to 2-months follow-up are reported in Table 2.

### Clinically significant improvement and reliable change

**Figure 4:**
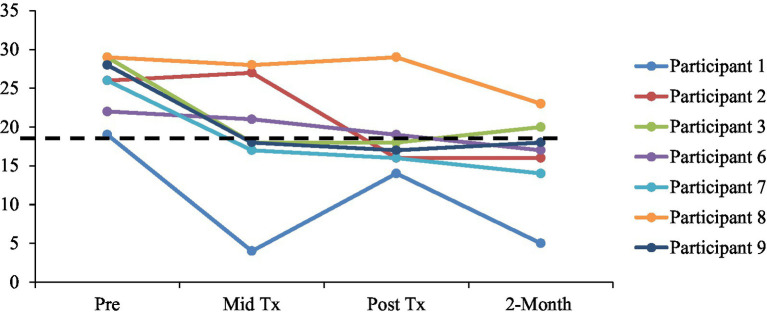
Clinically significant improvement on CY-BOCS (by scores below the line), defined by a reduction of two standard deviations from pre-treatment mean (<18.01). Tx = treatment.

The following analyses of clinical significance and reliable change are reported for treatment completers (*n* = 7). At post-treatment, 5 of the 7 children (71.4%) showed reliable change and clinically significant improvements in their symptom severity from pre-treatment as measured by the CY-BOCS total score (RCI cut-off = 4.19). At 2-month follow-up, all 7 (100%) children showed reliable change from pre-treatment, with 5 children (71.4%) continuing to meet criteria for clinically significant improvements on the CY-BOCS total scores. Figure 4 illustrates participants meeting clinically significant improvement on CY-BOCS.

In relation to diagnostic severity at post treatment, 3 of the 7 (42.9%) children were considered “improved” in a clinically significant way on the basis of diagnostic interviews (CSR; >2 SDs below pre-CSR mean). Furthermore, at 2-month follow-up, 4 of the 7 (57.1%) children also demonstrated clinically significant “improvement” on CSR ratings. Of the children who were not considered “improved” at post-treatment or follow-up, the diagnostic severity scores were reduced relative to pre-treatment, yet remained in the clinical range.

### Response and remission outcomes and comorbidity

In terms of treatment response rates, 57% (*n* = 4) of the sample were considered responders at post-treatment and 63% (*n* = 5) at follow-up. At post-treatment, there were no children in remission of OCD; however, at 2-month follow-up 13% (*n* = 1) had reached remission status.

In relation to diagnostic comorbidity, significant differences were observed in the total number of children’s diagnoses over time, *F*(2, 12) = 4.26, *p* = 0.040, *η_p_^2^* = 0.415. Although there was no significant reduction in the number of diagnoses from pre-treatment (*M* number comorbid diagnoses = 1.71, SD = 1.50) to post-treatment (*M* = 1.00; SD = 1.53); there were significant reductions in the number of comorbid diagnoses from pre-treatment to 2-month follow-up (*M* = 0.43, SD = 0.79), *t*(6) = 2.71, *p* = 0.035.

## Discussion

In an effort to address the major research-service treatment gap for pediatric OCD, this study developed and tested the preliminary efficacy of brief, telehealth delivered parent training in ERP. The treatment—FAST CBT (**F**amilies **A**ccessing **S**kills **T**raining in CBT-ERP) involved four modules that delivered ERP skills to parents of OCD-affected children *via* telehealth videoconference. This study utilized a multiple-baseline controlled design, supported by the evidence-based treatment movement (Task Force on Promotion and Dissemination, 1995) for the systematic evaluation of the efficacy of novel treatments in a controlled manner (Jarrett and Ollendick, 2012; Oar et al., 2015). The results of this study provide preliminary support for parent training in ERP *via* telehealth as an effective way to reduce symptoms for youth with OCD, with initial indications for whom this intervention might work best.

The results demonstrated that children experienced significant improvement across a broad range of measures, including clinician-and parent-rated symptom measures of OCD with significant gains made at post-treatment and follow-up, relative to pre-treatment assessment. Notably, there was stability in OCD target symptom severity across the baseline phase, providing within-subjects control for time. In addition, data analyzed at an individual level supported the effectiveness of the treatment, with 100% of the sample achieving reliable change, and 70% experiencing clinically significant improvements in OCD symptom severity. At post-treatment, 57% of the sample were considered responders, and this was maintained at 2-month follow-up, with 63% of children meeting responder status. These response results are conservative estimates of response relative to earlier treatment outcome studies, with the most recent consensus on response being defined as ≥35% reduction in CY-BOCS severity (Farhat et al., 2021) relative to earlier criteria which used ≥25% reduction.

However, it is notable that the remission rates in this study (11%) are much lower than those reported elsewhere (e.g., 49–51% at 6-month follow-up, (Farrell et al., 2022). Notably, the two children who did not reach clinically significant improvement were most severe (range of OCD). Thus, these data suggest that brief, parent training in CBT-ERP *via* telehealth provides an overall effective intervention that is likely to be of most benefit to children and youth who are mild to moderate in severity. However, given this study employed a shorter follow-up than those previously reported, it may be that effects of parent-training are amplified over time, with longer-term follow-up studies needed to examine this potential effect.

Closer examination of patterns across the single case data, highlight several individual differences in treatment outcomes. For example, Participant 4’s (P4) outcomes did not change across time. P4 withdrew from the study, so we utilized the last observation carried forward with case review method. Notably, P4 had a different expression of comorbidity compared to the other participants with a neurodevelopmental disorder and Tourette syndrome. This finding is consistent with past research that demonstrates that youth with OCD comorbid with ASD appear to have poorer treatment outcome (Griffiths et al., 2017). Another single case observation was with Participant 8 (P8) whose target symptoms became poorer across time. P8 was one of the oldest participants with more severe symptoms (CY-BOS). Research shows that parenting has stronger influence in younger children compared to adolescents who are more autonomous (Paikoff and Brooks-Gunn, 1991). This finding suggests that the current program might be more suited to younger children with milder symptoms and a shorter duration of illness.

There are limitations of the current study that warrant attention. Single-case designs require stability of symptoms over the baseline period, and while there was a general trend for stability, there was some fluctuation in parent-rated OCD target symptoms of participants during the baseline phase, which does reduce the causal inferences that can be drawn from the results of those participants. Parents may not be the most reliable rater’s of child and adolescent OCD symptoms, given the covert nature of obsessions and the often secretive or hidden nature of ritualizing. Future studies would be improved by using child and clinician ratings of symptoms for multiple-baseline design studies to improve reliability of symptom reporting. Furthermore, this design does not allow for a test of the novel intervention against another intervention, which would provide a more rigorous evaluation of a treatment, and as such, large RCTs of feasibility, acceptability and efficacy with active comparator conditions would strengthen the evidence for efficacy of this treatment. An additional limitation was that weekly baseline data was not collected weekly throughout the treatment phase of the design. This occurred due to the challenges of running the study remotely during COVID lockdowns, whereby we decided to reduce the amount of data being collected during the treatment phase to reduce burden on clinicians and participants. Furthermore, although the sample recruited was within the moderate to severe range of OCD and were highly comorbid increasing generalizability, the age range was limited to those aged 8 to 14 years, and the ethnicity of families was solely Caucasian, therefore limiting the generalizability of findings to more ethnically diverse samples, and with younger/older clients. Finally, the diagnostic raters, while independent and blind to previous assessment data and treatment information, were not blind to assessment time-point.

## Conclusion

Overall, the study provides favorable initial evidence for brief parent training in CBT-ERP delivered *via* telehealth for children and youth with OCD. The outcomes offer promise for more efficient models of treatment delivery and importantly, greater reach of EB ERP for this often severe and debilitating condition. The potential of this approach is that it may address the research-service treatment gap, and provide additional accessible, efficient and cost-effective means of families accessing specialist CBT-ERP treatment. Parent training in ERP, delivered *via* telehealth may be of most benefit to children and adolescents who present with mild to moderate OCD severity. Alternatively, it may be effective as a first line treatment for young people with a very recent onset of OCD; as a first line intervention in a stepped care or staged care model of intervention; as a parent-focused module to augment child-focused clinic-based treatment; or even as a preventative intervention for families with a strong familial history of OCD. This pilot study provides preliminary, yet promising outcomes associated with brief, parent-training in ERP *via* telehealth, which has the potential to address the research-service gap in the dissemination of exposure-based CBT for pediatric OCD.

## Data availability statement

The raw data supporting the conclusions of this article will be made available by the authors, upon request, without undue reservation.

## Ethics statement

The studies involving human participants were reviewed and approved by Griffith University Human Research Ethics Committee. Written informed consent to participate in this study was provided by the participants’ legal guardian/next of kin.

## Author contributions

LF, ES, and TO conceived and planned the study with other lead investigators (CD, AW, SoM, RW, and RS). NN, ShM, MM, RK, NB, and TM carried out the assessments and interventions. LF took the lead in writing the manuscript. LF, NN, ShM, MM, TM, CD, AW, SoM, NB, RK, GS, RW, RS, ES, and TO provided critical feedback and helped to shape the research, analysis and manuscript. All authors contributed to the article and approved the submitted version.

## Funding

This study was funded by the Griffith University Health Group capacity building scheme. Publication fees for this manuscript were supported by Virginia Tech Open Access Subvention Fund.

## Conflict of interest

ES discloses the following relationships: consultant for Biohaven Pharmaceuticals and Brainsway; Book royalties from Elsevier, Springer, American Psychological Association, Wiley, Oxford, Kingsley, and Guilford; Stock valued at less than $5,000 from NView; Research support from NIH, IOCDF, Ream Foundation, and Texas Higher Education Coordinating Board.

The remaining authors declare that the research was conducted in the absence of any commercial or financial relationships that could be construed as a potential conflict of interest.

## Publisher’s note

All claims expressed in this article are solely those of the authors and do not necessarily represent those of their affiliated organizations, or those of the publisher, the editors and the reviewers. Any product that may be evaluated in this article, or claim that may be made by its manufacturer, is not guaranteed or endorsed by the publisher.
